# Editorial

**Published:** 1861-03

**Authors:** 


### EDITORIAL.

Illinois State Medical Society.—We hope the profession throughout the State, will remember that the next regular meeting of the State Society will be held at Jacksonville, commencing on the first Tuesday in May. The place of meeting is attractive, and the objects to be accomplished important. We trust the profession in all parts of the State will be represented.

---

Medical College Commencements in this City.—*Rush Medical College.*—The eighteenth annual commencement of this institution was held in the college hall, on Wednesday evening Feb. 20th. The degree of M. D. was conferred by the President of the College faculty, Prof. Brainard, on *thirty-seven* candidates, as follows:

Allen S. Barnt, Charles Bunce, Wm. C. Brown, Sidney S. Buck, Benj. H. Bradshaw, Wilford Bates, Henry S. Blood, Daniel M. Cool, Elijah A. Clark, Thos. J. Dunn, Edw. C. DeForrest, M. M. Eaton, Henry W. Maynard, Richard E. McVoy, John Murphy, Samuel O. Owen, Allen M. Pierce, Henry V. Passage, Madison Ruce, Geo. Egbert, Wm. B. Graham, Henry J. Herrick, Zenas P. Hanson, Clinton D. Henton, John F. Kealdes, Ezekiel Keith, Enoch W. Keegan, Abner D. Kimball, Robt. M. Lackey, Z. James McMaster, James M. Mayfield, E. Fred Russell, Edward P. Talbot, Chas. B. Thompkins, Theodore W. Stull, Israel B. Washburne, O. G. Walker.

The honorary degree of M. D., was conferred upon Robert C. Hammill, Chicago, and Theodore Hoffman, Niles.

---

Medical Department of Lind University.—The second annual commencement of this college was held in the third Presbyterian Church on the evening of the 5th of March. A. large and attentive audience was present. The degree of M. D., was conferred by Prof H. A. Johnson, President of the College Faculty, on *twelve* candidates as follows :

J. M. Barlow, of Bellair, Ill. ; C. Dumreicher, of Chicago, Ill. ; F. S. C. Grayston, of Huntington, Ind. ; G. W. Morrill, of Shopiere, Wis. ; F. W. Reilly, of Chicago, Ill. ; Hiram Wanzer, of Chicago, Ill. ; T. J. Bluthardt, of Chicago, Ill. ; S. L. Fuller, of Chicago, Ill. ; O. A. Lewis, of Huntington, Ind. ; J. Nicolai, of S. Bend, Ind. ; D. W. Stewart, of Moline, Ill. ; H. T. Woodruff, Joliet, Ill.

The *ad-eundem* Degree of M. D., was conferred on Drs. D. C. Roundy and C. Miller, both of Geneva, Wis. The Honorary Degree of M. D. was conferred on Prof. T. Deville of England.

A premium was awarded to H. T. Woodruff of Joliet, Ill., for the best Inaugural Thesis ; another to Frank W. Reilly of Chicago, for the best Clinical Report ; and another to F. S. C. Grayston of Huntington, Ind., for the second best Clinical Report.

The Valedictory address was then delivered by Prof. M. K. Taylor of Galesburg. The address will be found in the present number of the Examiner, and will be read with interest and profit. After the exercises closed at the Church, the graduates and students, with the Faculty and guests repaired to the residence of Prof. N. S. Davis, where they enjoyed an entertainment and a social interchange of the most agreeable character.

---

Berkshire Medical Journal.—Edited by W. H. Thayer, M. D., of Keene, N. H., and R. Cresson Stiles, M. D., of Pittsfield Mass.

We have received the first two numbers of this new monthly periodical, and take pleasure in placing it on our exchange list. It is neatly printed and edited with ability.

Note.—The above was designed for the February number of the Examiner, but was accidently omitted. In the same number of the Examiner, under the head of Selections, were several pages of matter copied from the Berkshire Medical Journal, without being properly credited. Also a valuable article on Ergot copied from the Philadelphia Medical and Surgical Reporter, without credit. These omissions escaped our observation, by trusting the proof-reading of the selected matter to the publisher. We shall endeavor to be more careful in future.

---

Change of Name.—The New Orleans Medical News and Hospital Gazette, has been changed to *New Orleans Medical Times*; and has passed under the editorial management of Anthony Peniston, M. D.

---

New York Ophthalmic School.—The annual commencement of this institution was held in the fourteenth-street Medical College, in the presence of a large number of students and medical men. Peter Cooper occupied the chair. Dr. Stephenson, Senior Surgeon to the Institution, made a few preliminary remarks in regard to the organization and success of the institution, and concluded by awarding testimonials to the following gentlemen who composed the graduating class: J. Craig, Minn., G. W. Edwards, N. Y., C. F. Miesse, O., B. A. Watson, N. J., F. G. Stanley, M. D., Ill., J. E. Lynch, O.,

A. J. Harris, Penn., G. T. H. Scott, M. D. Iowa, W. L. Wheeler, N. Y., H. Hill, M. D., C. W., W. S. Souders, O., S. W. Briggs, M. D., N. B., J. L. Kiernan, M. D., N. Y., S. Ayers, N. J., H. S. Plympton, Mass., T. H. Stilwell, N. Y., W. R. Stilwell, N. Y., G. R. Wells, M. D. Wis., J. W. Robie, N. H., S. J. Dewey, M. D., Ill., A. D. Smith, Ga., C. R. Case, M. D., Mich., B. O. Reynolds, M. D., Wis., Calhoun Hill, N. C., R. V. M'Kim, M. D. Dr. J. P. Garrish, one of the Surgeons of the Hospital, delivered an excellent address to the students, and the Valedictory was spoken by Dr. James F. Kiernan, of the graduating class. The exercises were closed with a short address by Peter Cooper, who in the course of his remarks highly complimented the students connected with the Hospital, and in glowing terms eulogized the professors connected with the institution.

---

Summer Course of Medical Instruction.—The regular summer term of instruction in the Medical Department of Lind University, was commenced on Monday the 25th inst.; and will be continued until the first of October. A very respectable class of students were present, and have registered their names for the whole course.

---

Homœopathy and the Michigan University.—We learn from the Detroit Free Press, that the Legislature of Michigan, during its recent session, passed a law requiring the establishment of a chair of Homœopathy in the Medical Department of the State University, at Ann Arbor. It is claimed, however, and probably with truth, that the Legislature has no control over the subject; the constitution of the State having placed the regulation of the University and all its departments exclusively and fully under the charge of the Board of Regents. This board, only a few years since, after instituting a pretty thorough investigation in relation to the nature and claims of Homœopathy, refused to establish any such chair.

Whether the recent action of the Legislature will induce them to alter their former dicision, remains to be seen.

We think, however, that they will not be guilty of so great an act of folly.

### THE NEW SYDENHAM SOCIETY.

This is an English Society, instituted for the purpose of publishing standard works on Medical and Surgical subjects, at a low cost, so that they may be distributed among the largest possible number of the profession. The subscription is one guinea a year, ($5,25, U. S. currency,) which entitles a member to all the publications for the current year. The works are not obtainable in our bookstores, or in any way except by joining the Society. The following list indicates their character.

### THE PUBLICATIONS FOR 1859.

Vol. I.—*Diary* "On Syphilis in Infants and Children at the Breast." Translated by Dr. Whitley.

Vol. II.—*Gooch* "On the most important Diseases of Women and Children," with other Papers. Woodcuts. Prefactory Essay by Dr. Ferguson.

Vol. III. *Memoirs on Diphtheria.* From various French sources. Selected and translated by Dr. Semple. With a Bibliographical Appendix by Mr. Chatto.

Vol. IV. comprises the two Works of Professor *Schroeder Van-der Kolk*, (1st) "On the Spinal Cord," and (2nd) "On the Medulla Oblongata," and "On the Proximate Cause and Rational Treatment of Epilepsy." Translated by Dr. W. D. Moore, of Dublin. With numerous Lithographs.

Vol. V. contains translations of the following Monographs:—

1st. *Kussmaul* and *Tenner's* "Experimental Researches on the Effects of Loss of Blood in inducing Convulsions." Translated by Dr. Bronner, of Bradford.

2nd. *Wagner* "On the Resection of Bones and Joints." Translated by Mr. T. Holmes. Numerous Woodcuts.

3rd. Professor *Graefe's* Three Papers on Glaucoma, Iridectomy, &c. &c. Translated by Mr. T. Windsor, of Manchester.

THE PUBLICATIONS FOR 1860.

Vol. VI.—*Clinical Memoirs on Abdominal Tumours and Intumesence.* By Dr. Bright. Collected and reprinted from the Guy's Hospital Reports- Edited by Dr. Barlow. With numerous Woodcuts.

Vol. VII.—*A Year-Book for* 1859.—The Year-book is designed to form a Register in condensed abstract of all Important Communications whether British or Foreign, in Medicine, Surgery, and their allied Sciences during the year. It will consist of five sections:—1st. Anatomy and Physiology. Edited by Dr. Harley. 2nd. Medicine. Edited by Dr. Handfield Jones. 3rd. Surgery. Edited by Mr. Hulke. 4th. Diseases of Women and Children. Edited by Dr. Graily Hewit. And 5th. Forensic Medicine and Toxicology. Edited by Dr. Odling.

Vol. VIII.—*Frerich's Clinical Account of Diseases of the Liver.* Vol. I. With Woodcuts. Translated by Dr. Murchison.

Vol. IX.—*Vogel and Neubauer* on the Examination of the Urine. A Manual intended for the assistance of the practical physician. With Woodcuts and Plates.

Vol. X.—*Hebra's Atlas of Illustrations of Skin Diseases.*

The works for 1860, and probably those for 1859, may yet be had by subscribing for those years. The Subscription for 1861 is now due, and should be paid in advance, to secure the publications of the year. Thus pre-payment is essential to the efficient and prompt action of the Society in publishing the books, and relieves from embarrassing uncertainty. As it is a mutual benefit association, it is hoped that all members of the profession will take interest enough in it to mention it to their friends. The expenses of exchange and carriage are divided among the local members. The circulars of the Society may be obtained, and a specimen of the books seen at the office of Dr. V. L. Hurlbut, 122 Randolph Street, or at the office of the undersigned who is authorized to forward subscriptions.

CHAS. G. SMITH, M. D.,
**95 Washington St. Chicago.**

OBITUARY—DR. JOHN W. FRANCIS.—In the decease of Dr. John W. Francis—he died at 3 A. M. yesterday, at his residence in East Sixteenth street—New York has lost a marked and shining celebrity. His services to the profession he passionately loved and adorned are at an end; his genial face has faded from mortal view; his eloquence of anecdote, his rare and grand speculations, his noble and hearty benevolence, are no longer living and loved realities. His death was not expected. Though nearly 72 years old, he seemed hale and hearty. Afflicted with carbuncles on the back, he submitted last week to an operation which he thought very much relieved him. The draft on his physical energies proved too great. Suddenly, and without pain, he passed away, retaining his senses to the last.

John Wakeman Francis was born in this city, on the 17th of November, 1789. His father came from Germany, his mother was a Philadelphian, of Swiss descent. At an early age, John was placed in the printing office of George Long, in this city. His ambition soared above type-setting, and he early showed a fondness for books, a love for scholastic pursuits. Employing his leisure time in study, he proved himself an apt as well as a vigilant scholar. His genius was recognized, his industry rewarded. Judicious friends came to the aid of the young and striving student, and in 1807 he was enroled to enter the junior class of Columbia College, at the same time commencing the study of medicine under the celebrated Dr. Hosack. In 1809 he graduated, and at once gave his undivided attention to his medical studies. In 1810, in conjunction with Dr. Hosack, he commenced the publication of the "Amercan Medical and Philosophical Register," both acting jointly as editors. In 1811 he recieved his degree of M. D. from the New York College of Physicians and Surgeons—his was among the first degrees granted by this College—and the same year he was admitted to partnership with Dr. Hosack, a partnership which continued through nine years. In 1813 he was appointed to the chair of Materia Medica in his *alma mater*.—*N- Y. Times.*

